# Chitosan-Based Therapeutic Systems for Superficial Candidiasis Treatment. Synergetic Activity of Nystatin and Propolis

**DOI:** 10.3390/polym14040689

**Published:** 2022-02-11

**Authors:** Andra-Cristina Humelnicu, Petrișor Samoilă, Corneliu Cojocaru, Raluca Dumitriu, Andra-Cristina Bostănaru, Mihai Mareș, Valeria Harabagiu, Bogdan C. Simionescu

**Affiliations:** 1 “Petru Poni” Institute of Macromolecular Chemistry, 41A Grigore Ghica Voda Alley, 700487 Iasi, Romania; humelnicu.andra@icmpp.ro (A.-C.H.); samoila.petrisor@icmpp.ro (P.S.); cojocaru.corneliu@icmpp.ro (C.C.); rdumi@icmpp.ro (R.D.); bcsimion@icmpp.ro (B.C.S.); 2 Laboratory of Antimicrobial Chemotherapy, Faculty of Veterinary Medicine, “Ion Ionescu de la Brad” Iasi University of Life Sciences (IULS), 8 Mihail Sadoveanu Alley, 700489 Iasi, Romania; mycomedica@gmail.com

**Keywords:** chitosan, drug delivery systems, nystatin, propolis, antifungal activity

## Abstract

The paper deals with new approaches to chitosan (CS)-based antifungal therapeutic formulations designed to fulfill the requirements of specific applications. Gel-like formulations were prepared by mixing CS dissolved in aqueous lactic acid (LA) solution with nystatin (NYS) powder and/or propolis (PRO) aqueous solution dispersed in glycerin, followed by water evaporation to yield flexible mesoporous (pore widths of 2–4 nm) films of high specific surfaces between 1 × 10^3^ and 1.7 × 10^3^ m^2^/g. Morphological evaluation of the antifungal films showed uniform dispersion and downsizing of NYS crystallites (with initial sizes up to 50 μm). Their mechanical properties were found to be close to those of soft tissues (Young’s modulus values between 0.044–0.025 MPa). The films presented hydration capacities in physiological condition depending on their composition, i.e., higher for NYS-charged (628%), as compared with PRO loaded films (118–129%). All NYS charged films presented a quick release for the first 10 min followed by a progressive increase of the release efficiency at 48.6%, for the samples containing NYS alone and decreasing values with increasing amount of PRO to 45.9% and 42.8% after 5 h. By in vitro analysis, the hydrogels with acidic pH values around 3.8 were proven to be active against *Candida albicans* and *Candida glabrata* species. The time-killing assay performed during 24 h on *Candida albicans* in synthetic vagina-simulative medium showed that the hydrogel formulations containing both NYS and PRO presented the faster slowing down of the fungal growth, from colony-forming unit (CFU)/mL of 1.24 × 10^7^ to CFU/mL < 10 (starting from the first 6 h).

## 1. Introduction

More than one billion people are affected by superficial *Candida* infections as oral candidiasis and vaginitis (vulvovaginal candidiasis) [1,2]. An improper treatment of these infections can lead to the fungi spreading from the surface of the body to the internal organs (kidneys, heart, brain) and to the blood, causing deadly invasive infections or candidemia [3]. Worldwide, the incidence of invasive *Candida* infections is increasing (700,000 cases annually), and is associated with considerable mortality [2,4]. The antagonistic augmentation of case numbers and geographical spread by the end of 2020 [5] requires an increased effort from scientists to promote appropriate prevention, protection and therapeutic systems, and to combat candidiasis in early stages. 

The treatment of superficial candidiasis involves the development of mucoadhesive pharmaceutical systems for the local administration of antifungal agents. The polysaccharide-based therapeutic systems are known for achieving specific functions in a complex biological environment, since they are considered one of the most propitious subjects lying on the frontier between chemistry, biology, medicine, and bioengineering [6]. Undoubtedly, natural polymers are promptly recognized and embraced by the human body considering their biochemical similarity with extracellular matrix components [7]. Chitosan is a cationic amino-polysaccharide which possesses useful biological properties, such as mucoadhesion, antibacterial and antifungal activity, antioxidant, hemostatic, and antiseptic characteristics [8]. The reactive hydroxyl and amino groups on the chitosan chain facilitate different reactions with other active compounds and are able to perform a multitude of intermolecular and intramolecular interactions [9]. Moreover, chitosan-based therapeutic systems are known for their capacity to incorporate and release multiple active principles, either simultaneously or sequentially, for achieving more efficient associated therapy [10]. Therefore, this study is based on the well-known mucoadhesive properties of chitosan, but also on its resistance in contact with the oral and vaginal mucosa [11,12]. 

Among the few classes of active principles with antifungal effect (azoles, polyenes, echinocandins, allylamines, and pyrimidine analogs) [13,14], emphasis was put on polyenes characterized by a large spectrum of activity induced by their unusual mechanism of action. By comparison with other classes of antifungals, polyenes do not target a specific enzyme, but rather interact selectively with sterols (especially ergosterol) in the plasma membranes of fungi, thus causing loss of membrane function, altered permeability and nutrient damage [15]. Nystatin is a broad-spectrum polyene antifungal agent derived from *Streptomyces noursei*, known for increased susceptibility of the fungi and for its high efficacy rate in the prophylaxis and the treatment of superficial candidiasis [16,17].

In terms of antifungal efficiency, among the use of nystatin, this work aims to evaluate certain bioactive compounds derived from natural sources, namely propolis—a resinous mixture produced by the honey bees, also known for its antifungal activity—and to scrutinize the combination therapy [18]. The novelty of the approach consists in providing chitosan film formulations with mechanical properties close to those of the soft tissues, by taking advantage of chitosan increased flexibility and bioadhesivity induced by lactic acid pH regulator and of the plasticizing properties of both glycerin additive and propolis bioactive agent. Moreover, to the best of our knowledge, this work is the first to demonstrate the combined effect of nystatin and propolis aqueous solution against *Candida* species, when administered together, under relevant simulative conditions, on vulvovaginal candidiasis. Thus, chitosan-based therapeutic formulations containing either propolis or nystatin, either their mixture were prepared as hydrogels and films, and proved to be active in the treatment of vulvovaginal and oral candidiasis, respectively.

## 2. Materials and Methods

### 2.1. Materials 

Chitosan (CS), with an average molar mass of 290 kDa and 81.6% deacetylation degree (as previously determined by viscometry, respectively by NMR analysis [19]) was provided by Merck Chemical (Saint Louis, MO, USA). L-(+)-Lactic acid (LA) and anhydrous glycerin (Gly) were purchased from Chemical Company (Iași, Romania). Nystatin (NYS) drug, internationally qualified by USP Reference Standard, with particle sizes under 50 µm (Figure S1 in Supplementary Materials) was supplied by Antibiotice SA, Iași, Romania. Aqueous Propolis 30% (*w*/*v*) (PRO) with 7.72 mg/mL polyphenols and 0.26 mg/mL flavones/flavonoids contents (spectrophotometrically determined according to Singleton et al., 1999 [20] at APHIS-DIA Laboratory, Cluj-Napoca, Romania) was purchased from Dapis Transilvania (Cluj-Napoca, Romania). All the analytical grade chemicals were used as received. *Candida albicans* ATCC 90028 and *Candida glabrata* ATCC 90030 were provided by American Type Culture Collection (Manassas, VA, USA). Yeast Nitrogen Base Agar with dextrose and Yeast Extract Peptone Dextrose Agar (YPD) were purchased from Merck Chemical (Saint Louis, MO, USA), while Sabouraud dextrose agar (SDA) was acquired from Biokar Diagnostics (Allonne, France).

### 2.2. Preparation of Chitosan Antifungal Therapeutic Formulations

Chitosan based hydrogels or flexible films of different contents of antifungal compounds (Table 1) were prepared in several steps, as described below. First, appropriate solutions or dispersions of each individual component were obtained. Thus, a chitosan stock solution of 3% (*w*/*v*) concentration was prepared by dissolving the biopolymer in a 2% (*w*/*v*) lactic acid solution under continuous stirring at 600 rpm and 40 °C for 24 h. NYS (15 mg) was dispersed in glycerin (0.4 g) by magnetic stirring of their mixture for 30 min, at 40 °C and 400 rpm, and a subsequent homogenization by ultrasonication (Emmi 12 HC ultrasonic bath, 100% ultrasonic efficiency) for 10 min. PRO aqueous solution (0.3 mL) was also mixed with Gly (0.4 g) under stirring at 40 °C and 400 rpm for 30 min.

CS-based formulations charged either with NYS (CS-NYS) or with PRO (CS-PRO) were obtained by adding 10 mL of the stock solution of chitosan over each of their mixtures with Gly and stirring for 1 h at room temperature.

Two chitosan formulations, CS-NYS-PRO1 and CS-NYS-PRO2, containing both antifungal agents in different ratios (PRO/NYS = 3/1 or 6/1 *w*/*w*) were prepared by subsequently adding calculated amounts of PRO aqueous solution and of 10 mL of the stock solution of chitosan onto NYS-Gly dispersion (previously prepared as described above) and stirring at room temperature till homogenization was achieved (about 1 h). All the prepared hydrogels were characterized by pH values of 3.85 ± 0.02, as measured by using a HANNA instruments HI8417pH meter (Amorim, Portugal).

An identical set of hydrogels was separately prepared and each sample was poured into a 5 cm diameter Petri dish. The drying at room temperature yielded transparent CS-NYS, CS-PRO, CS-NYS-PRO1 and CS-NYS-PRO2 films of micrometric thickness (measured with a Dial Thickness Gauge 7301 handheld micrometer—Mitoyuto Corporation, Kangagawa, Japan; accuracy of 1 µm), depending on composition (Table 1). For analytical purposes, uncharged chitosan hydrogel and film (CS-LA) were also prepared.

### 2.3. Methods of Characterization

#### 2.3.1. Structural and Morphological Characterization of Film Formulations

Fourier transform (FTIR) spectra of film formulations were registered by using a Bruker Vertex 70 spectrophotometer (Bruker Optics, Ettlingen, Germany), in ATR (Attenuated Total Reflectance) mode (wavelength range of 4000–600 cm^−1^, resolution of 2 cm^−1^ and 64 scans at room temperature). Scanning electron microscopy (SEM) images of cross-sections of the prepared films were obtained on an FEI QUANTA 200 electron scanning microscope (Brno, Czech Republic) with a resolution of 4 nm at 30 kV. The drug dispersion in the films was visualized on a Leica Microsystems Polarized Optical Microscope (Wetzlar, Germania).

#### 2.3.2. Mechanical Properties of the Films

Tensile strength, elongation at break, and Young’s modulus were determined on an Instron 3365 equipment (Norwood, MA, USA) with two columns and a 500 N force cell. Dumbbell-shaped samples for each film were cut using a press (length/width/active length = 50/4.1/35 mm) and the uniaxial stress–strain curves of the samples occurs at 50 mm/min elongation speed. The stress (σ_b_) and strain (ε_b_) at break were calculated according to Equations (1) and (2), respectively:

σ_b_ (MPa) = F_b_/A (1)

ε_b_ (%) = Δl/l_0_ × 100 (2)

where F_b_ is the breaking force, A is the cross-sectional area of the sample at time t, l_0_ represents the initial length, and ∆l represents the elongation at traction. Based on the specific deformation curve, the modulus of elasticity for each film was also determined as the ratio between the stress and the tensile deformation (1%).

#### 2.3.3. Swelling Behavior of Film Formulations in Simulated Conditions

1 cm^2^ of film samples were dried in an oven at 40 °C till constant weight, were immersed in 50 mL phosphate buffered saline (PBS) solution of pH 7.4 and were placed in an Orbital Shaker-Incubator ES-20/60 (Biosan—Riga, Latvia) at 37 °C and 80 rpm. At predetermined time intervals, the samples were removed from the immersion medium; the excess PBS solution was removed from the surface by buffering with filter paper and the samples were weighed. The kinetics of the film swelling was evaluated by a gravimetric method, according to Equation (3):

S_t_ (g/g) = (w_t_ − w_0_)/w_0_ (3)

where S_t_ (w/w) represents the value of swelling capacity at time t (min), w_0_ is the initial mass of the dried sample, and w_t_ is the mass of the sample at time t after immersion in PBS. The data were plotted by using the average values of the three determinations for each film formulation. To establish the equilibrium swelling capacity, the experimental data were also analyzed by applying a pseudo-second order kinetic model (PSO), expressed by Equation (4) [21,22]:

S_t_ (g/g) = k_s_ × S_e_^2^/(1+ k × S_e_ × t) (4)

where S_t_ (g/g) represents the swelling capacity at contact time t (min), k_s_ is the constant of the swelling rate and S_e_ represents the theoretical swelling capacity at equilibrium time. 

The swelling dynamics (diffusion-controlled or relaxation-controlled) of the films into PBS up to 5 h was evaluated using the Equation (5) [23,24], adapted after Korsmeyer and Peppas (K-P) model [25]:

F = S_t_/S_e_ = k_p_ × t^n^ (5)

where F is the swelling fraction, S_t_ and S_e_ are the swelling capacities at time t, and at equilibrium, respectively; k_p_ is a constant dependent on the polymeric network; n is the diffusion parameter of aqueous PBS in the formulation film [23]. 

The diffusion parameter value is characterizing the diffusion mechanism of aqueous molecules in the film formulations: n < 0.5 implies a Fickian diffusion-controlled mechanism; 0.5 < n < 1 suggests anomalous non-Fickian diffusion; n = 1 indicates a relaxation-controlled water transport and n > 1 represents a supercase II of diffusion [22]. The experimental data were processed by using SCILAB 6.1.0 software. 

#### 2.3.4. In Vitro Nystatin/Propolis Release from Film Formulations

Pre-weighed dried samples of antifungal chitosan hydrogels were immersed each in 50 mL of neutral PBS solution (pH 7.4), placed in an Orbital Shaker-Incubator ES-20/60 (Biosan—Riga, Latvia) and maintained at 37 °C and 80 rpm. At predetermined time intervals, 0.5 mL were extracted from each solution (subsequently being replaced with the same volume of initial buffer solution) and were spectrophotometrically analyzed using a double-beam UV-VIS spectrophotometer Hitachi U-3900 (Hitachi High-Tech Europe GmbH—Krefeld, Germania). The drugs concentrations in solution were established based on previously performed calibration curves and the release efficiency was evaluated as a function of time. Nystatin characteristic absorption bands were identified at 293, 305, and 320 nm. The calibration curves were plotted and the concentrations were determined by using the band at 320 nm for nystatin, and the specific absorption band at 284 nm for propolis.

In order to determine the drugs release mechanism (Fickian or non-Fickian diffusion), the experimental data were mathematically fitted (SCILAB 6.1.0 software) using the semi-empirical equation (Equation (6)) proposed by Korsmeyer and Peppas (1981), which describes both in vitro drug release from thin plane films and the stability behavior [25]:

M_t_/M_∞_ = k × t^n^ (6)

where M_t_/M_∞_ refers to the fractional drug release at time t; M_t_ and M_∞_ are the total amount of drug released at time t and at infinite time, respectively; k represents the transport constant, n is the diffusion exponent that indicates the type of drug release transport (Quasi-Fickian diffusion for n < 0.5, Fickian diffusion for n = 0.5, non-Fickian transport for n > 0.5, Case II transport for n = 1, and supercase II transport for n > 1) [26].

#### 2.3.5. Dynamic Vapor Sorption Measurements

The water vapor sorption capacity of the hydrogel formulations was determined in dynamic regime by using a fully automated gravimetric device IGAsorp provided by Hiden Analytical (Warrington, UK). The samples were placed in a special container and dried at 25 °C using a nitrogen flow of 250 mL/min until their weights reached equilibrium at a relative humidity, RH, less than 1%. Subsequently, the RH was gradually increased from 0 to 90%, with humidity steps of 10% (at pre-established equilibrium time between 40–60 min) and the sorption equilibrium was registered for each step in the sorption curve. The RH was further decreased and desorption curves were also registered. To evaluate the specific surface area, Brunauer–Emmett–Teller kinetic model (BET) was applied by modeling the sorption isotherms registered under dynamic conditions, according to Equation (7) [27,28]:

W = W_m_ × C × RH/(1 − RH)(1 − RH + C × RH) (7)

where W and W_m_ represent the weight of absorbed water and the weight of water forming a monolayer, respectively; C is the sorption constant and RH represents the relative humidity. 

The average pore size, r_pm_, was estimated by applying Barrett, Joyner, and Halenda model (BJH) [28], based on calculation methods for cylindrical pores, in accordance with Equations (8) and (9):

V_liq_ = n/100 × ρ_a_ (8)

r_pm_ = 2V_liq_/A (9)

where V_liq_ is the liquid volume; n is the absorption percentage; ρ_a_ represents the adsorbed phase density and A is the specific surface area determined by BET method.

#### 2.3.6. Rheological Properties of the Hydrogels

Rheological measurements were performed on a Physica MCR 301 Stress Controlled Rotational Rheometer (Anton Paar Company, Graz, Austria) with cone-plate geometry (50 mm diameter, an angle of 1°/99 μm truncation). The viscoelastic properties were analyzed based on the dynamic oscillatory and temperature sweep measurements. Preliminary tests were performed at 10 rad/s in the 0.1–200% deformation range with a constant shear displacement γ of 10%. The dynamic oscillatory tests were conducted at constant temperature of 35 ± 0.1 °C (accurately controlled by Peltier heating system) and angular frequency (ω) in the range of 0.05/0.1–100 rad/s. The elastic modulus, G′, which represents the solid-like component of viscoelastic behavior of the material, the viscous modulus, G″, representative for liquid-like component were investigated as functions of the angular frequency ω (rad/s).

The viscoelastic behavior was evaluated also by determining the tangent of δ phase angle (Equation (10)) and the complex viscosity (Equation (11)), which is related to complex modulus as described by Equation (12) [29]:

tan δ = G″/G′(10)

η* = G*/ω(11)

G* = G′ + iG″(12)

where δ is the phase angle (a relative measure for the visco-elastic properties); G′ and G″ are the elastic and viscous moduli; η* is the complex viscosity and G* represents the complex modulus, which is a complex number with i representing an imaginary number [30].

Temperature sweep measurements were realized at a constant shear rate of 10 s^−1^, over the temperature range 5–40 °C with a heating rate of 2 °C/min. After loading and before measurement, a 5 min rest time for each sample was allowed to ensure stress relaxation and temperature equilibration. The viscosity of the hydrogels (η) was evaluated as a function of temperature and the activation energy (Eη) of the viscous flow was calculated by fitting the experimental data with Arrhenius exponential equation (Equation (13)), using a logarithmic form according to Equation (14). The natural logarithm of η was plotted as function of 1/T (K^−1^) [31]:

η = A exp (Eη/RT)(13)

ln η = ln A + Eη/RT(14)

where η is viscosity of the hydrogels; A represents the Arrhenius factor (a constant associated with the nature of the liquid); Eη is the viscous flow activation energy; R is the molar gas constant (1.9872 cal·K^−1^·mol^−1^) and T is the temperature (K). 

#### 2.3.7. Antifungal Activity of the Hydrogel

The prepared chitosan formulations (CS-NYS, CS-PRO, CS-NIS-PRO1 and CS-NIS-PRO2) as well as CS-LA solution were evaluated for antifungal activity against two reference strains from the American Type Culture Collection (*Candida albicans* and *Candida glabrata*), which are the main pathogens responsible for invasive candidiasis. Both *Candida* strains were stored in 20% glycerin at −80 °C. Prior to testing, each strain was refreshed on Sabouraud dextrose agar (SDA) and incubated for 48 h at 30 °C. The yeast strains were used when a maximal number of conidia were formed.

The antifungal activity of CS-based hydrogels was evaluated by using the agar disk diffusion method. Microbial suspensions were prepared in sterile saline solution to obtain an optical turbidity comparable to that of the 0.5 McFarland standards (each suspension contains 1 × 108 colony-forming units/mL (CFU/mL)). Volumes of 0.2 mL from each inoculum were taken and spread onto Yeast Nitrogen Base Agar with dextrose previously poured in Petri dishes. After the drying of the medium surface, each sample of chitosan hydrogels (10 µL) was added. The antifungal properties of the tested hydrogels were determined by measuring fungal growth inhibition, under standard conditions (after incubation for 48 h at a temperature of 30 °C). All experiments were performed in triplicate, in order to verify the final results. The diameter of the inhibition zone around the hydrogel samples was finally measured using a caliper with digital display.

#### 2.3.8. Time–Kill Assay

Time–kill experiments were performed on Candida albicans ATCC MYA-2876 (SC5314 wild type) using a slightly modified previously described method [32]. Thus, a synthetic vagina-simulative medium (SVSM) was prepared [33,34] to assure specific biomimetic conditions. Before performing the tests, the strain was sub cultured at least twice and grown for 24 h at 35 °C on SDA plates.

To examine the rate of killing, from each strain, a 5 McFarland suspension in SVSM was prepared and adjusted to 1.24 × 10^7^ CFU/mL using the TC20 automated cell counter (Bio-rad, Hercules, CA, USA). Subsequently, equal volumes of yeast cell suspension and chitosan hydrogels dispersions in SVSM were mixed and incubated at 36 ± 1 °C, to obtain final mixtures for each compound. A drug free control was also prepared by mixing equal volumes of yeast suspension and SVSM. At predetermined time intervals (0, 6, 12, and 24 h), 1000 µL aliquot from each test and control tube was serially diluted in sterile distilled water, plated onto YPD agar medium and incubated 48 h at 36 ± 1 °C, in order to evaluate the number of CFU/mL. The reproducible detection limit for colony counts is 10^1^ CFU/mL. All time–kill curves were plotted for the binary mixture (log10 CFU/mL against time) and the assays were conducted in duplicate and on two separate occasions.

## 3. Results and Discussions

### 3.1. Therapeutic Formulations Design and Preparative Protocol

Hydrogels and films formulations are considered to be the most suitable therapeutic systems for the treatment of superficial candidiasis, as they can cover a larger area for the administration of antifungal agents, offering, at the same time, physical protection [35]. Although, chitosan-based hydrogels with propolis and nystatin (among others active principles) were evaluated by Perchyonok et al. (2012–2014) as restorative materials for increasing the dentin bond strength capacity and for oral mucositis treatment [35,36,37,38], this work is a first report on evaluating and demonstrating the antifungal effect of nystatin and/or propolis loaded chitosan systems under relevant simulative conditions on vulvovaginal candidiasis. In addition, the use of L-lactic acid for chitosan solubilization and of an aqueous propolis extract in the preparation of chitosan-based nystatin and/or propolis delivery systems are for the first time investigated.

Glycerin was selected for nystatin dispersion and as an additive in antifungal formulations, having in mind its ability to solubilize lipophilic drugs in w/o emulsions, to promote local delivery of the drugs in topical and mucosal applications [39], as well as to act as a plasticizer for chitosan [40,41].

The dissolution of chitosan was performed in lactic acid solution, as it has been shown to induce to the CS formulations mechanical properties suitable for their use on soft tissues, by increasing flexibility (for the films) and bioadhesion (for hydrogels), as opposed to acetic acid [42]. Although DL-lactic acid was used in most pharmaceutical applications, we choose to use L-(+)-lactic acid, primarily because it is a known compound of human metabolism and secondly because it was proven to lower the tensile strength and to increase elongation at break of chitosan films [43]. The physicochemical characterization of film formulations (mechanical properties, swelling and drug release in simulative medium) was done to demonstrate their usefulness as buccal drug delivery systems. As the chitosan hydrogels were characterized by pH values of 3.85 ± 0.02, close to the value of vaginal fluids (pH = 4.2 [33]), they were tested as therapeutic systems under simulative conditions in the therapy of vulvovaginal candidiasis.

### 3.2. Structural Characterization

The structure of nystatin (NYS), its ^13^C NMR and ^1^H NMR spectra are given in Supplementary Materials, Figures S2 and S3, proving the high purity of the polyene macrolide [44,45,46]. The structure of chitosan and a comparison between the FTIR spectra of pristine chitosan powder and of the film prepared by its dissolving in aqueous lactic acid solution and drying are presented in Supplementary Materials, Figures S4 and S5, respectively. Polyphenols and flavonoids (not structurally identified) are present in propolis (PRO) solubilized in aqueous media, in the proportion mentioned in the experimental part, as specified by the supplier.

**Figure 1:**
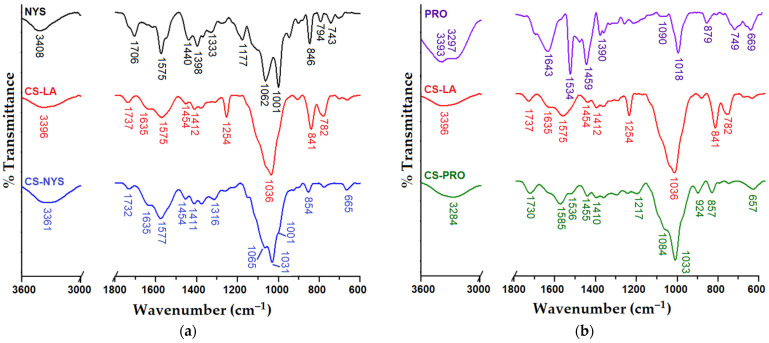
FTIR spectra of the individual components and: CS-NYS film (**a**); CS-PRO film (**b**).

Figure 1 compares the characteristics FTIR absorptions of the drugs (NYS and PRO), CS-LA matrix and of the resulted film formulations (CS-NYS and CS-PRO, as typical exemples). As no visible modifications were found in the region of asymmetric and symmetric C–H stretching vibrations (2900–2800 cm^−1^), for the sake of clarity, this region is not represented in Figure 1. NYS (Figure 1a) is characterized by a large number of hydroxyl groups whose stretching vibrations are found in the range 3200–3700 cm^−1^, overlapping with the vibrations of the NH_2_ group. Other characteristic bands are found at 1706 cm^−1^ (C=O stretching vibrations from ester and carboxylic acid groups), at 1575 cm^−1^ and 846 cm^−1^ (typical vibrations corresponding to the polyenes C=C units), in the intervals 1440–1320 cm^−1^ (deformation vibrations of CH_3_) and 1180–1000 cm^−1^ (vibrations of the C–C–O and C–O–C groups, respectively) [47,48].

Propolis (PRO) chemical composition is well-known for polyphenols and flavonoids content, with characteristic absorption bands clearly evidenced in the FTIR spectrum (Figure 1b). Wide absorption band centered at 3395 cm^−1^ is attributed to the OH stretching vibration of phenolic compounds and to the formation of inter(intra)molecular hydrogen bonds. Specific to PRO, the following absorption bands are identified: 1643 cm^−1^ (C=O flavonoid carbonyl stretching vibration), 1534 cm^−1^, and 1459 cm^−1^ (aromatic rings of polyphenols and C=C groups of flavonoids) [49,50], 1390 cm^−1^ (C–O stretching vibrations and O-H deformation, specific to phenols), 1018 cm^−1^ (strong absorption band of C–O esters asymmetric stretching vibration), 879 cm^−1^, 749 cm^−1^, and 669 cm^−1^ (medium absorption bands corresponding to the out-of-plane deformation vibration of phenol CH, and CH_2_ rocking of hydrocarbons) [50,51].

The chitosan film obtained from its aqueous solution (CS-LA) reveals the absorption band at 1737 cm^−1^ due to the carboxylate (COO^–^) groups provided by lactic acid and a broad band centered at 1575 cm^−1^ that was attributed to the deformation vibrations of protonated amine groups (superimposed over amide vibrations), mainly confirming the electrostatic interactions between protonated chitosan amine groups and carboxylate ions (NH_3_^+ –^OOCCH(OH)CH_3_) [52]. The presence of lactic acid determines the appearance of the band at 1454 cm^−1^, associated with the CH_3_ asymmetric deformation vibrations in its structure [53]. Moreover, the absorption band centered at 1036 cm^−1^ indicates the presence of stretching vibrations of the C–O groups of lactic acid, overlapped with the C–O–C vibrations of chitosan [54]. The asymmetric C–O stretching vibrations are found at 1254 cm^−1^, while the C–C stretching and C=O deformation vibrations from the lactic acid structure appear at 841 cm^−1^ and 782 cm^−1^ [55]. The lack of a new amide absorption band at about 1540 cm^−1^ indicates the absence of covalently grafting of LA on CS [56].

FTIR spectra of the antifungal films of nystatin (CS-NYS) and propolis (CS-PRO) embedded into chitosan matrix can be observed in the bottom of the Figure 1a,b, respectively. Due to the structural similarities, the characteristic bands of nystatin and/or propolis are overlapping with polysaccharide bands. In addition, the low content of the drugs into the CS formulations makes rather difficult their identifications in the spectra of the corresponding formulations. However, an in-depth comparison between the spectra of the intermediates and of the corresponding film formulations reveals the presence of the drugs into CS matrix. Thus, as a consequence of the increased number of OH groups coming from NYS and PRO components, and of the larger diversity of hydrogen bonding, the typical broad O–H absorption bands in the region 3600–3200 cm^−1^ have higher intensities in the spectra of both CS-NYS and CS-PRO film formulations, as compared to that of CS-LA matrix, and are shifted at different wavenumbers, as compared with the spectra of the individual components. The embedment of the active principles in the polysaccharide matrix induced a slight displacement of the characteristic C=O lactate band from 1737 (CS-LA) to 1732 cm^−1^ (CS-NYS) and 1730 cm^−1^ (CS-PRO). Moreover, some of the drug bands are also visible in the spectra of the corresponding film formulations. Thusly, CS-NYS spectrum shows broader and a more intense band at 1577 cm^−1^ due to the superposition of the C=C band of NYS located at 1575 cm^−1^ over amide/amine bands of CS; the bands at 1062 and 1001 cm^−1^ of NYS are visible as shoulders (1065 and 1001 cm^−1^) in the region of the C–O–C vibrations of CS-NYS spectrum. Similar traces of PRO are visible in the spectrum of CS-PRO film (e.g., the appearance of a shoulder at 1536 cm^−1^ induced by the contribution of the aromatic ring vibrations of polyphenols and the displacement of the amide/protonated amine band of CS from 1575 cm^−1^ to 1585 cm^−1^).

### 3.3. Films Morphology

**Figure 2:**
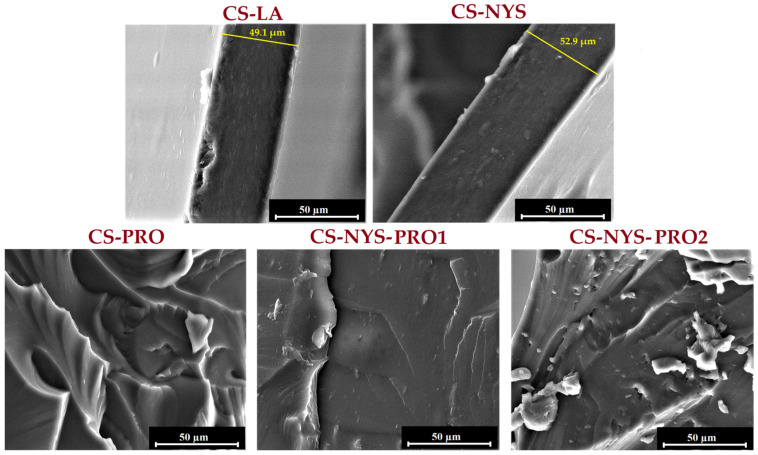
Scanning electron microscopy images of the cross-sections of CS-LA, CS-NYS, CS-PRO, CS-NYS-PRO1 and CS-NYS-PRO2 films.

The morphology of chitosan antifungal films (CS-NYS, CS-PRO, CS-NYS-PRO1, and CS-NYS-PRO2) was examinated by reference to CS-LA film on SEM images of their cross-sections (Figure 2). The CS-LA sample presents a homogeneous, dense and cohesive structure, characteristic for unmodified chitosan films. The addition of nystatin to the polysaccharide matrix (CS-NYS) did not induce major morphological changes of the film. However, besides the dense and compact morphology, the dispersion of NYS as white nanoparticles in the chitosan matrix can be distinguished. The downsizing of NYS crystallites (with initial dimensions up to 50 μm as seen in Figure S1, Supplementary Materials), as an effect of ultrasonication in glycerin and incorporation into the chitosan matrix can be noticed. The introduction of propolis into the chitosan matrix induces film thickening, as compared to CS-LA and CS-NYS, and visible morphological changes. Most are evidenced for CS-PRO and CS-NYS-PRO2 samples, due to the higher amount of propolis. Thus, CS-PRO presents an homogenous wavy organized structure, while CS-NYS-PRO2 shows a discontinuous morphology with phase separation between chitosan and glycerin/propolis plasticizers. Similar morphology was observed when sorbitol was used as a plasticizer for chitosan films [57].

**Figure 3:**
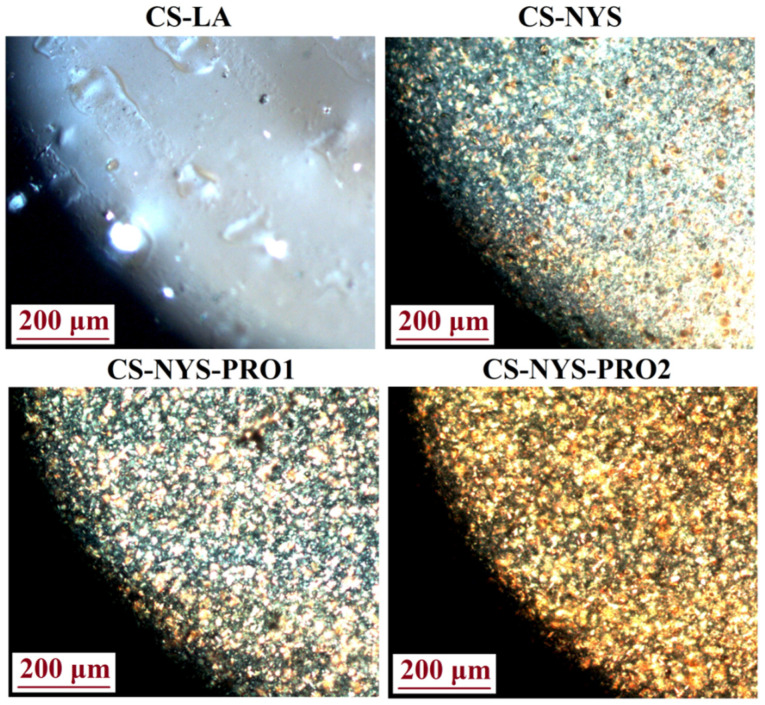
Polarized light microscopy images of CS-LA, CS-NYS, CS-NYS-PRO1 and CS-NYS-PRO2 films surface.

The morphology of the prepared transparent films was also investigated by polarized light microscopy (Figure 3). The image obtained for CS-LA film is typical for a partially crystalline polymer with crystalline domains embedded in the amorphous ones. Even if nystatin is known for its insolubility in aqueous medium and its tendency to form aggregates, the uniform dispersion of the polyene in the CS-NYS, CS-NYS-PRO1 and CS-NYS-PRO2 chitosan films is easily visible.

### 3.4. Mechanical Properties of Films

Chitosan with nystatin and/or propolis hydrogels were dried and the mechanical properties of the resulted micronic films (see the thickness in Table 1) were evaluated in order to prove potential similarities with the mechanical properties of the human soft tissues and the suitability of their application in the treatment of oral candidiasis. Mechanical properties of the antifungal films were investigated based on the characteristic stress–strain curves (Figure S6 in Supplementary Materials). The tensile strength at break (σ_b_), elongation at break (ε_b_) and modulus of elasticity (Y) were determined and evaluated by comparison with the those of CS-LA film (Table 2). All charged chitosan films are much ductile, contrary to CS-LA sample. They present more than 10 folds and about 2 order of magnitude decrease of the tensile strenght (from 73.3 MPa to 3.0–8.6 MPa) and of the Young’s modulus (from 9.310 to 0.044–0.025 MPa), respectively, as well as a considerable increase of the elongation at break (from 7.7 to 68.9–113.9%). These characteristics of the charged CS films are mainly due to the plasticizing action synergetically induced by GLY and PRO to the CS matrix. Similar behavior was reported by others for Gly-CS mixtures and was explained by the formation of hydrogen bonds between the OH groups of the triol compound and the protonated amino groups (NH^3+^) of the polysaccharide, leading to the reorganization of chitosan chains and formation of an orderly structure [40,41]. NYS alone provided to the film the highest elasticity (the higher elongation at break was registered for CS-NYS film), while PRO seems to provide most pronounced plasticizing effect (lower values of the three studied mechanical properties were registered for all PRO containing films). Propolis was also proposed as a bioplasticizer for starch [51]. All the prepared antifungal chitosan films are characterized by increased elasticity, malleability and mechanical properties close to those of the soft tissues (Young’s modulus of soft biological tissues has values lower than <1 MPa [58]), being suitable for application on buccal mucosa.

### 3.5. Swelling Capacity of Films

**Figure 4:**
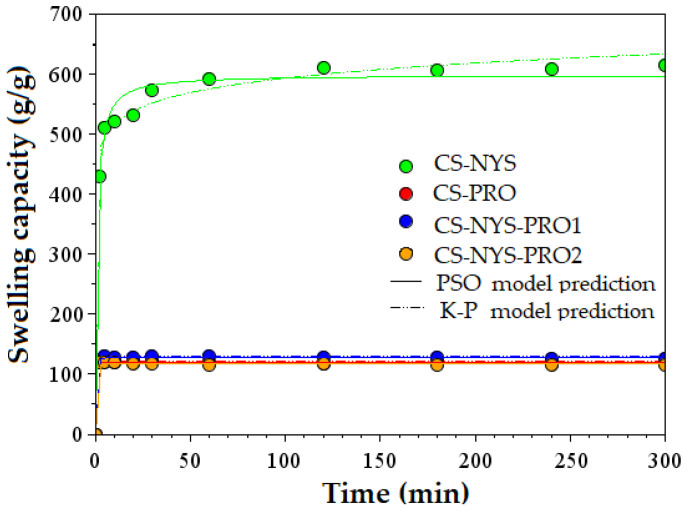
Swelling kinetics of chitosan films with nystatin (CS-NYS), propolis (CS-PRO), and both nystatin and propolis (CS-NYS-PRO1, CS-NYS-PRO2).

Knowing that the excessive hydration of the films might lead to decreased bioadhesion at the interface with buccal mucosa [59], the swelling capacities of chitosan films charged with nystatin and/or propolis were evaluated in PBS, at physiological oral pH 7.4 and 37 °C. It should be noted that the pure chitosan film (CS-LA) was completely dissolved in the aqueous medium, while all chitosan charged films were recovered after 24 h contact with PBS solution as self-standing swollen hydrogels. Their increased hidrodynamic stability can be explained by the effect of the electrostatic interactions and/or intermolecular hydrogen bonding between polysacharide and the drug molecules. Figure 4 shows the 5 h swelling kinetics of the charged CS films and the plots for PSO and K-P kinetic models. For CS-NYS sample a rapid increase of the swelling capacity to more than 500% the first 2 min, followed by an importand decrease of the swelling rate was registered. The film reaches swelling degrees of 618 and 751% after 5 and 24 h, respectively of immersion in PBS (Table S1).

CS films charged with propolis (CS-PRO, CS-NYS-PRO1 and CS-NYS-PRO2) presented much lower capacities of welling, as compared to CS-NYS sample, probably due to the chemical crosslinking of the polyphenols found in the natural antifungal agent to the amino groups of the chitosan. All these three samples indicated almost identical swelling behavior, with an immediate (first 2 min) increase to practical maximum swelling capacity, between 117 and 122% (Table S1). 

The kinetic parameters of the PSO and K-P models are given in Table S1 for all drug charged CS films. The theoretical equilibrium swelling capacity (S_e_) values are close to those of the experimental swelling capacity after 5 h, except the CS-NYS sample. The diffusion exponents, n, have values lower than 0.5, suggesting a Fickian diffusion-controlled swelling kinetics, with the diffusion of the water molecules in the polymeric matrix faster than the chitosan chains relaxation [22,24].

### 3.6. In Vitro NYS and PRO Release from Antifungal Films

**Figure 5:**
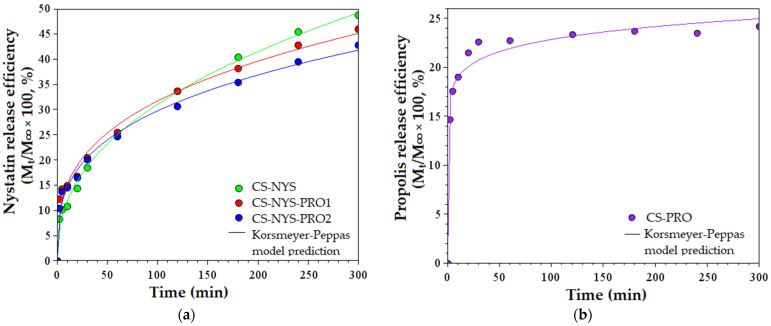
Release efficiency of: (**a**) Nystatin from CS-NYS, CS-PRO-NYS1 and CS-PRO-NYS2; (**b**) Propolis from CS-PRO; the experimental data were fitted based on Korsmeyer and Peppas kinetic model.

Nystatin and propolis release efficiencies (%) were calculated based on their concentrations in PBS imersion solutions over time (24 h), as determined from their UV-VIS spectra (Figure S7, Supplementary Materials). The release of the drugs from the antifungal films during 5 h is graphically represented in Figure 5. As one may see from Figure 5a, all NYS charged films presented a quick release the first 10 min and progressively increasing release efficiency up to 48.6% (CS-NYS), 45.9% (CS-NYS-PRO1), and 42.8% (CS-NYS-PRO2) after 5 h. Further, the NYS release continues to rise by 5–7% at 24 h (Table S2). The adding of propolis to CS-NYS formulation induced a lower NYS release efficiency, as the crosslinking between polyphenols and chitosan makes more difficult the diffusion of NYS through the resulted polymeric network (CS-NYS-PRO2 had higher propolis content and lowest release efficiency).

Due to the superposition of the absorptions of NYS on the characteristic UV-VIS band of PRO, the release efficiency of the later was represented for CS-PRO film only (Figure 5b). PRO release curve shows a burst effect the first 30 min (with a release efficiency of 22.58%), followed by a plateau located at about 24.2%, which lasts at the same value after 24 h. 

Korsmeyer and Peppas kinetic release model was applied for fitting the in vitro NYS and PRO release experimental data (Figure 5). For NYS, the model provided a good fitting, indicating that the release mechanism is based on diffusion through the polysaccharide matrix. The fitting of PRO release experimental data showed a good fitting the first 20 min, followed by a satisfactory fitting up to 5 h. By calculating kinetics parameters of the semi-empirical matehmatical model (given in Table S2), it was established that the mechanims of both antifungal agents transport is based on Quasi-Fickian diffusion (n < 0.5) and the release mechanism occurs from non-swellable matrix diffusion [26].

### 3.7. Dynamic Vapor Sorption onto the Hydrogels

The behavior of the hydrogel formulations in the presence of moisture was investigated and the water vapor sorption/desorption isotherms were represented in Figure S8 (Supplementary Materials). Based on the IUPAC classification the isotherms are related to Type V curves, specific to water sorption on hydrophobic micro- and mesoporous surfaces and indicate weak adsorbent-water interaction [27]. Similar isotherms were registered for other chitosan-based hydrogels [60,61].

The hydrogels containing both NYS and PRO presented enhanced water vapor barrier properties, with lower water sorption capacity values (89.8 for CS-NYS-PRO1 and 88.1% CS-NYS-PRO2) as compared to the uncharged CS-LA sample (94.1%) as given in Table S3. This may be explained by the simultaneous effects of hydrophobic NYS component and of chemical crosslinking of CS amino groups by polyphenols, which makes more difficult the diffusion of water vapors through the polymeric network. This behavior is supported by the results of swelling capacity of the films formulations (Figure 5). 

The BET and BJH models were applied to evaluate the specific surface areas and the pore dimensions, respectively (Table S3, Supplementary Materials). The values obtained indicate the presence of larger mesopores for not charged CS-LA samples (width about 14 nm) as compared to NYS and PRO loaded samples (pore widths between about 2 and 4 nm). Moreover, the specific surface areas increased several times from 275 m^2^/g for CS-LA sample to 1035, 1379, 1444 and 1727 m^2^/g for CS-PRO, CS-NYS, CS-NYS-PRO1, and CS-NYS-PRO2 samples, respectively, as an effect of both the nature and content of functional groups and the pore size over the complex mode of the sorption capacity of the studied samples. 

### 3.8. Rheological Properties of Chitosan Hydrogel Formulations

**Figure 6:**
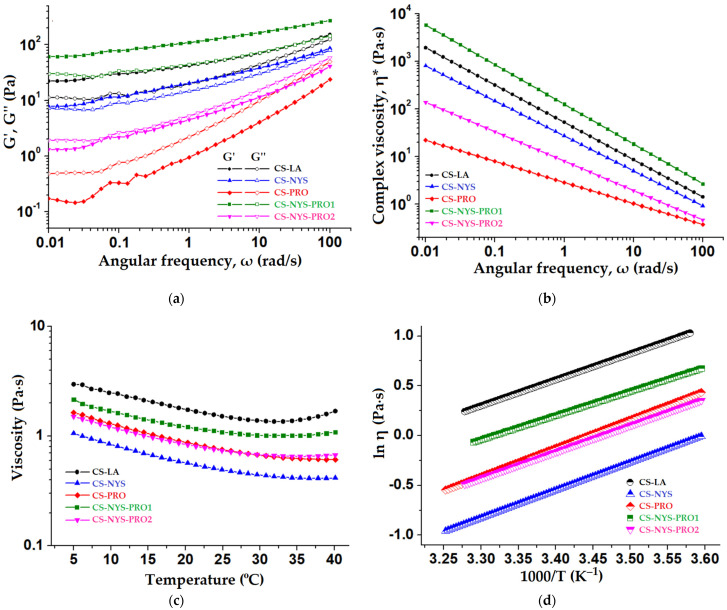
Rheological properties of chitosan hydrogel formulations: (**a**) dynamic viscoelastic moduli G′ (filled symbols) and G″ (open symbols) dependence on angular frequency at constant temperature of 35 °C and γ of 10%; (**b**) complex viscosity (η*) as a function of angular frequency (35 °C and γ = 10%); (**c**) temperature influence on the viscosity of hydrogel formulations; (**d**) Arrhenius fitting of the experimental data.

The rheological properties of chitosan hydrogels (with and without active principles) were evaluated as functions of angular frequency or temperature (Figure 6). Preliminary deformation tests at 0.1–200% deformation range (γ of 10%) confirmed that tests are in the linear viscoelastic regime (LVE). The dynamic oscillatory measurements were performed at 35 °C (temperature close to that of physiological body temperature of 37 °C) and the properties such as elasic modulus (G′), viscous modulus (G″) and complex viscosity (η*) were investigated in the range of 0.01–100 rad/s.

To establish the type of the hydrogels, the evolution of G′ and G″ at 35 °C and a γ=10% vs. ω is represented in Figure 6a and their values at ω = 1 Hz are given in Table S4, Supplementary Materials. All the hydrogels are characterized by increasing G′ and G″ moduli values with increasing angular frequency. As seen from this figure, the CS-LA, CS-NYS and CS-NYS-PRO1 hydrogels present a solid-like behavior (G′ modulus higher than G″ modulus), which denotes a polymeric network structure that is self-dependent of the applied rheological stress parameters. On the contrary, the hydrogels with higher content of propolis (CS-PRO and CS-NYS-PRO2) exhibit a predominantly liquid-like behavior (G″ > G′), indicating properties of a more viscous than elastic polymeric network. The rheological behavior of the hydrogels was also verified by the calculation of the loss tangent (tan δ) as represented in Figure S9, Supplementary Materials. The subunitary values of the tan δ confirms the gel like structure of the CS-LA, CS-NYS and CS-NYS-PRO1, while values higher than 1 of the loss tangents are found for the liquid-like behavior of the CS-PRO and CS-NYS-PRO2 hydrogels (Table S4, Supplementary Materials).

All chitosan-based hydrogels presented a linear decrease of the complex viscosity (η*) with the increase of the angular frequency (Figure 5b) induced by the breaking of the intermolecular forces and by the alignment of chitosan chains in the same direction with the applied stress. This behavior was observed by others in cationic hydrogel composites based on synthetic polymers and suggests a non-Newtonian behavior for all the range of angular frequencies, with a shear-thinning behavior [29]. 

The temperature sweep measurements were performed in the range of 5 to 40 °C, as it was proved that the storage temperature of chitosan solution is favorable at 5 °C as it reduces the polyssacharide decomposition [62] and the viscosity (η) of the hydrogels was evaluated up to the human body temperature (37 °C). Figure 6c shows an expected trend of decreasing viscosity of chitosan hydrogels when temperature increases, due to the augmentation of the polymeric thermal motion. However, all the samples present increased viscosity for temperatures exceeding 35 °C (more evident for CS-LA), mostly because of the moisture loss when subjected to higher temperatures [63].

The activation energy (Eη) for the viscous flow was calculated from the regression results obtained by Arrhenius fitting of the temperature-dependence curve of the viscosity (Figure 6d). Close to each other and positive activation energy (Eη) values (Table S4, Supplementary Materials) were registered for all hydrogels, as expected for solutions with thermo-thinning behavior (viscosity decrease with temperature increase).

### 3.9. Antifungal Activity of Hydrogel Formulations

**Figure 7:**
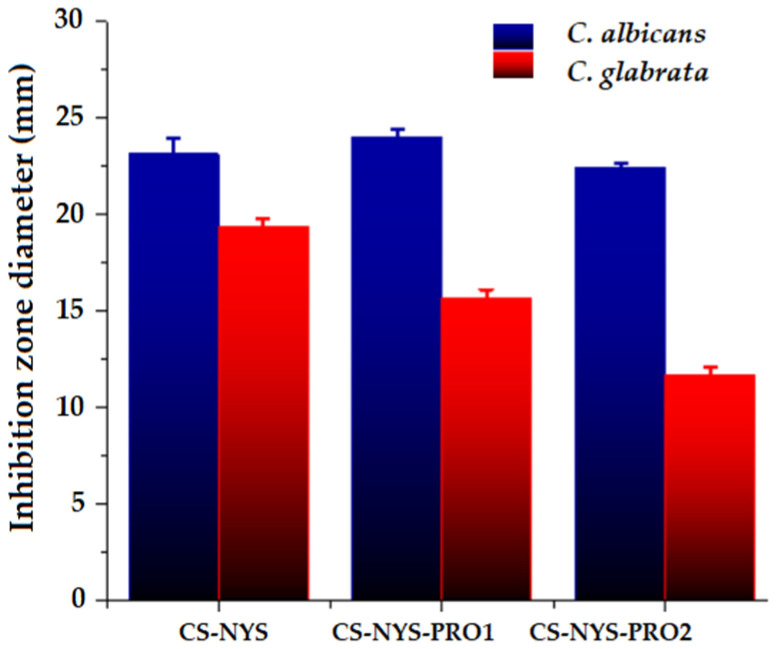
Diameters of the inhibition zones in the antifungal activity test of chitosan hydrogels against Candida albicans and Candida glabrata; error bars represent standard deviation (for n = 10).

The in vitro antifungal activity was investigated against two Candida species (Candida albicans and Candida glabrata) on hydrogels samples, using the agar disk diffusion method which supposes the addition of the hydrogels on the culture medium pre-inoculated with the microbial suspension, and measuring the clear zone caused by fungal growth inhibition around the hydrogels, after 48 h of incubation. The images of the inhibition zones and the average inhibition diameters are given in Figure S10, Supplementary Materials, and Figure 7, respectively. All the hydrogels killed the fungal cultures when placed in direct contact, but only chitosan hydrogels with NYS content (CS-NYS, CS-PRO-NYS1, CS-PRO-NYS2) presented inhibition of the fungal growth on a large area around the hydrogels, for both analyzed Candida strains. However, CS-NYS-PRO1 had the highest inhibition zone diameter (24 mm) compared with CS-NYS and CS-NYS-PRO2 (23, and 22 mm respectively) when evaluated against Candida albicans, which denotes the cumulative antifungal effect of NYS and PRO. The highest inhibition growth diameter against Candida glabrata was obtained for the CS-NYS hydrogel (19 mm), while CS-NYS-PRO1 and CS-NYS-PRO2 had diameters of 16, and 12 mm, respectively.

### 3.10. Time–Kill Assay Test

**Figure 8:**
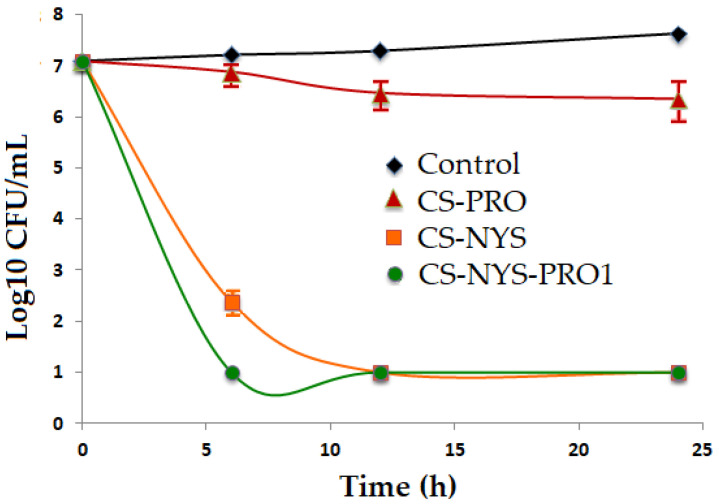
Time–kill curves for evaluation of the fungicidal activity of CS-NYS, CS-PRO and CS-NYS-PRO1 against yeast cells of *Candida albicans* as compared to the control assay; the error bars represent standard deviation for time killing kinetics (n = 4).

The time–kill kinetics against the Candida albicans (Figure 8) was assessed for the hydrogels that presented higher antifungal activity (CS-NYS and CS-NYS-PRO1), as compared with the hydrogel with less antifungal inhibition (CS-PRO) and a control sample. The time–kill assay supports the results previously obtained in agar plate antifungal testing against C. albicans, showing that CS-PRO-NYS1 exhibit the strongest and faster slowing down of the fungal growth (from the first 6 h). A similar trend is identified also for the chitosan hydrogel charged with nystatin, which reveal fungal killing efficiency < 10^1^ CFUs/mL in the first 12 h (Table S5). Corroborating data, it could be appreciated that the under-study chitosan hydrogels with nystatin and propolis have a potent fungicidal effect with a fungal burden reduction of 6-log killing (99.999% killing and 10 fungi left) after 24 h.

## 4. Conclusions

CS dissolved in aqueous lactic solution was demonstrated by structural (FTIR) and morphological (SEM, polarized light microscopy) investigations to successfully embed nystatin and/or propolis to yield gel-like or flexible film formulations. The antifungal films presented increased elasticity and mechanical properties close to those of soft tissues (Young’s modulus lower than 1 MPa), adequate hydration capacities to maintain films bioadhesion and a progressive release of nystatin based on Quasi-Fickian diffusion mechanism, being suitable for application on buccal mucous membranes (cheek mucosa). As established by in vitro antifungal analysis, the samples containing higher amounts of propolis (CS-PRO and CS-NYS-PRO2) do not show noticeable activity, contrary to CS-NYS and CS-NYS-PRO1 (lower amount of PRO) samples, which proved to be efficient against *C. albicans* and *C. glabrata*. The results were confirmed by time–kill assay in synthetic vagina-simulative medium, where the hydrogel containing both NYS and smaller amounts of PRO (CS-PRO-NYS1) proved a combined effect on combating *C. albicans*, exhibiting the strongest and faster slowing down of fungal growth from the first 6 h of contact. Further studies are ongoing to demonstrate the additive or synergistic effect of these two active principles against candida species.

## Figures and Tables

**Table 1:** Composition of chitosan formulations * and the thickness of the films.

Hydrogel/Film Code	NYS (mg)	PRO (mL Solution/mg Solid Compound)	Films Thickness (µm)
CS-LA	-	-	49
CS-NYS	15	-	53
CS-PRO	-	0.30/90	210
CS-NYS-PRO1	15	0.15/45	166
CS-NYS-PRO2	15	0.30/90	220

* all hydrogel formulations also contain 0.3 g CS (in 10 mL 2% LA solution) and 0.4 g Gly; the films were obtained by hydrogel drying and each formulation has a composition identical to the corresponding hydrogel, without water.

**Table 2:** Mechanical properties * of CS films.

Film Code	σ_b_ (MPa)	ε_b_ (%)	Y (MPa)
CS-LA	73.3	7.7	9.310
CS-NYS	8.6	113.9	0.044
CS-PRO	7.5	88.1	0.031
CS-NYS-PRO1	5.4	74.8	0.036
CS-NYS-PRO2	3.0	68.9	0.025

* tensile strength at break—σ_b_; elongation at break—ε_b_; Young’s modulus—Y.

## Data Availability

Not applicable.

## Supplementary Materials

The following supporting information can be downloaded at: https://www.mdpi.com/article/10.3390/polym14040689/s1, Figure S1: Nystatin powder: (**a**) Scanning electron microscopy images for nystatin; (**b**) particle size distribution histogram (particle sizes were determined using NIH Image J software); Figure S2: ^13^C-RMN spectrum of nystatin; IUPAC Name: (1S,3R,4R,7R,9R,11R,15S,16R,17R,18S,19E,21E,25E,27E,29E,31E,33R,35S,36R,37S)-33-[(2R,3S,4S,5S,6R)-4-amino-3,5-dihydroxy-6-methyloxan-2-yl]oxy-1,3,4,7,9,11,17,37-octahydroxy-15,16,18-trimethyl-13-oxo-14,39-dioxabicyclo[3 3.3.1] nonatriaconta-19,21,25,27,29,31-hexaene-36-carboxylic acid); signal attributions made according to ref. [44,45,46]; Figure S3: ^1^H-RMN spectrum of nystatin. IUPAC Name: 1S,3R,4R,7R,9R,11R,15S,16R,17R,18S,19E,21E,25E,27E,29E,31E,33R,35S,36R,37S)-33- [(2R,3S, 4S,5S,6R)-4-amino-3,5-dihydroxy-6-methyloxan-2-yl]oxy-1,3,4,7,9,11,17,37-octahydroxy- 15,16,18-trimethyl-13-oxo-14,39-dioxabicyclo[33.3.1]nonatriaconta-19,21,25,27,29,31-hexaene-36-carboxylic acid); signal attributions made according to ref. [44,45,46]; Figure S4: Chemical formula of chitosan with highlighting the functional groups: free primary amino groups (NH_2_ to C2), primary hydroxyl group (OH to C6) and secondary hydroxyl groups (OH to C3); *m* and *n* represent the numbers of deacetylated (d-glucosamine) and N-acetyl-d-glucosamine repeating units, linked by (1–4)-β-glycosidic linkages, respectively; Figure S5: FTIR spectra of chitosan powder (CS) and chitosan gel (3%) after dispersion in 2% lactic acid solution (CS-LA); Figure S6: Characteristic stress-strain curves of the films: (**a**) without biological active compounds (CS-LA); (**b**) nystatin, propolis, and nystatin/propolis loaded chitosan films (CS-NYS, CS-PRO, CS-NYS-PRO1, CS-NYS-PRO2); Table S1: Parameters of pseudo-second order (PSO) and Korsmeyer–Peppas (K-P) kinetic models, where k_s_ is the constant of the swelling rate, S_e_ is the theoretical swelling capacity at equilibrium, k_p_ is a constant dependent on the polymeric network; n is the diffusion parameter of aqueous PBS in the formulation film and SD represents the standard deviation of n = 11); swelling capacity in PBS of chitosan charged films after 5 and 24 h; Figure S7: Evolution in time of UV-VIS spectra of: nystatin released from CS-NYS (**a**), CS-NYS-PRO1 (**b**) and CS-NYS-PRO2 (**c**); propolis released from CS-PRO (**d**); Table S2: NYS and PRO release from chitosan films: Korsmeyer-Peppas kinetics parameters (k represents the transport constant, n is the diffusion exponent and SD is standard deviation of n = 11), and release efficiency after 5 and 24 h.; Figure S8. Sorption/desorption isotherms for the hydrogel formulations; Table S3. Surface parameters of the hydrogels evaluated based on adsorption/desorption isotherms: water vapor sorption capacity, final weight (W); average pore size (r_pm_) and BET data (surface area and monolayer weight); Figure S9: The loss tangent (tan δ = G″/G′) as a function of angular frequency; Table S4: Viscoelastic parameters (G′, G″ and tan δ) at a frequency of 1 Hz and viscous flow activation energy (Eη) calculated by fitting the temperature-dependence curve of the viscosity by Arrhenius equation; Figure S10. Antifungal activity of chitosan hydrogels against: (**a**) *C. albicans* and (**b**) *C. glabrata*; Table S5: Killing-time efficiency of CS-NYS, CS-PRO and CS-NYS-PRO1 hydrogels, against *Candida albicans* in 24 h as compared to the control test.
