# Supplementary material for: Chitosan-Based Therapeutic Systems for Superficial Candidiasis Treatment. Synergetic Activity of Nystatin and Propolis
Source: Polymers (Basel). 2022 Feb 11;14(4):689. doi: 10.3390/polym14040689 (PMC8876245; doi:10.3390/polym14040689)
Supplement: Supplementary file 1 [file polymers-14-00689-s001.zip › polymers-1571628-supplementary-done.pdf]

polymers

## Supplementary materials

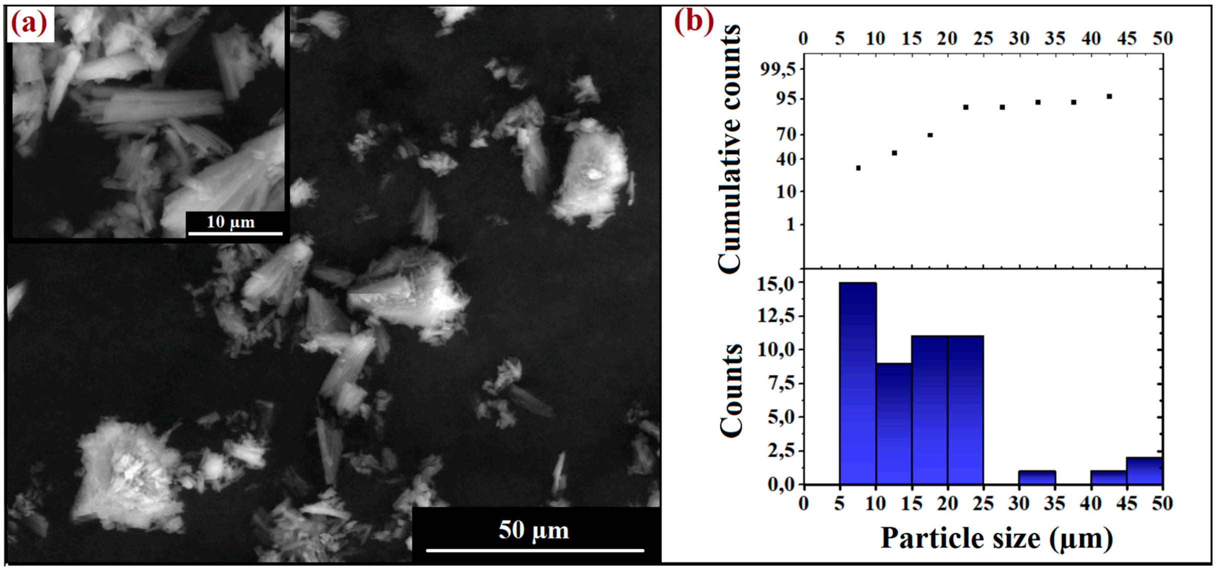


**Figure S1.** Nystatin powder: (**a**) Scanning electron microscopy images for nystatin powder and (**b**) particle size distribution histogram (particle sizes were determined using NIH Image J software).

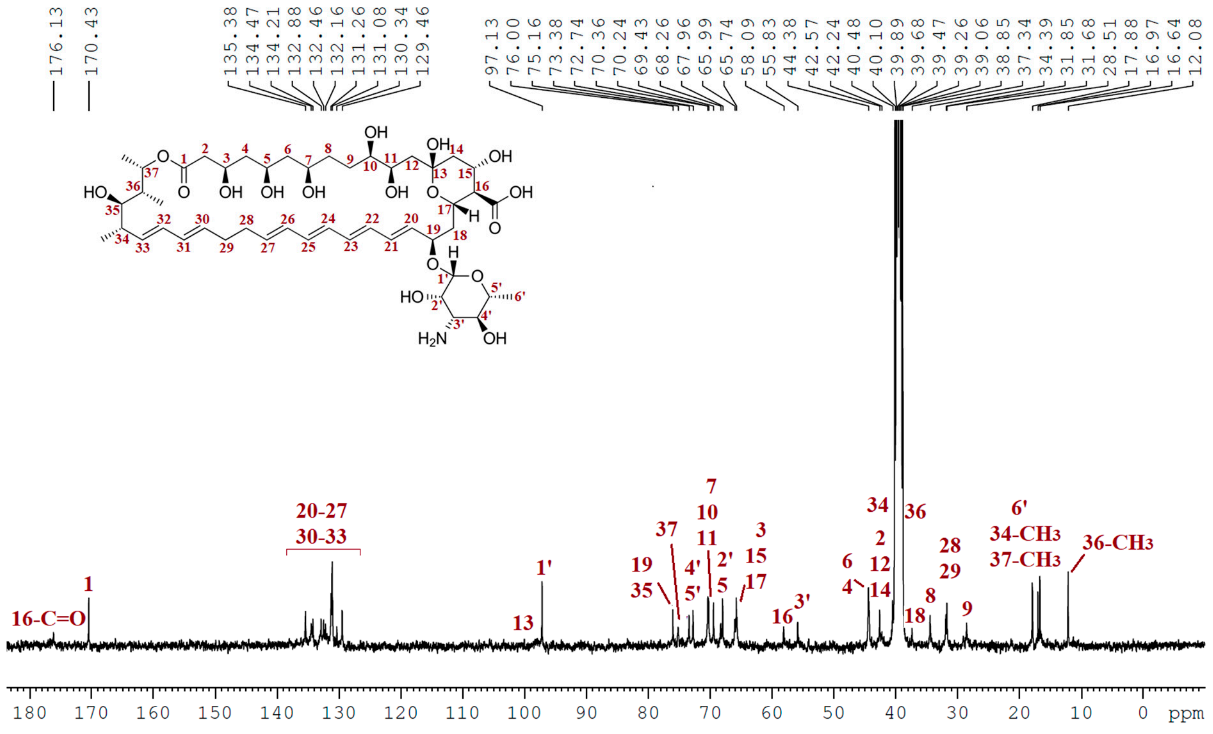


**Figure S2.** $^{13}$C-RMN spectrum of nystatin; IUPAC Name:(1S,3R,4R,7R,9R,11R,15S,16R,17R,18S,19E, 21E,25E, 27E, 29E,31E,33R,35S,36R,37S)-33-[(2R,3S,4S,5S,6R)-4-amino-3,5-dihydroxy-6-methyloxan-2-yl]oxy-1,3,4,7,9,11,17,37-octahydroxy-15,16,18-trimethyl-13-oxo-14,39-dioxabicyclo[33.3.1]nonatriaconta-19,21,25,27,29,31-hexaene-36-carboxylic acid); signal attributions made according to ref. [44-46].

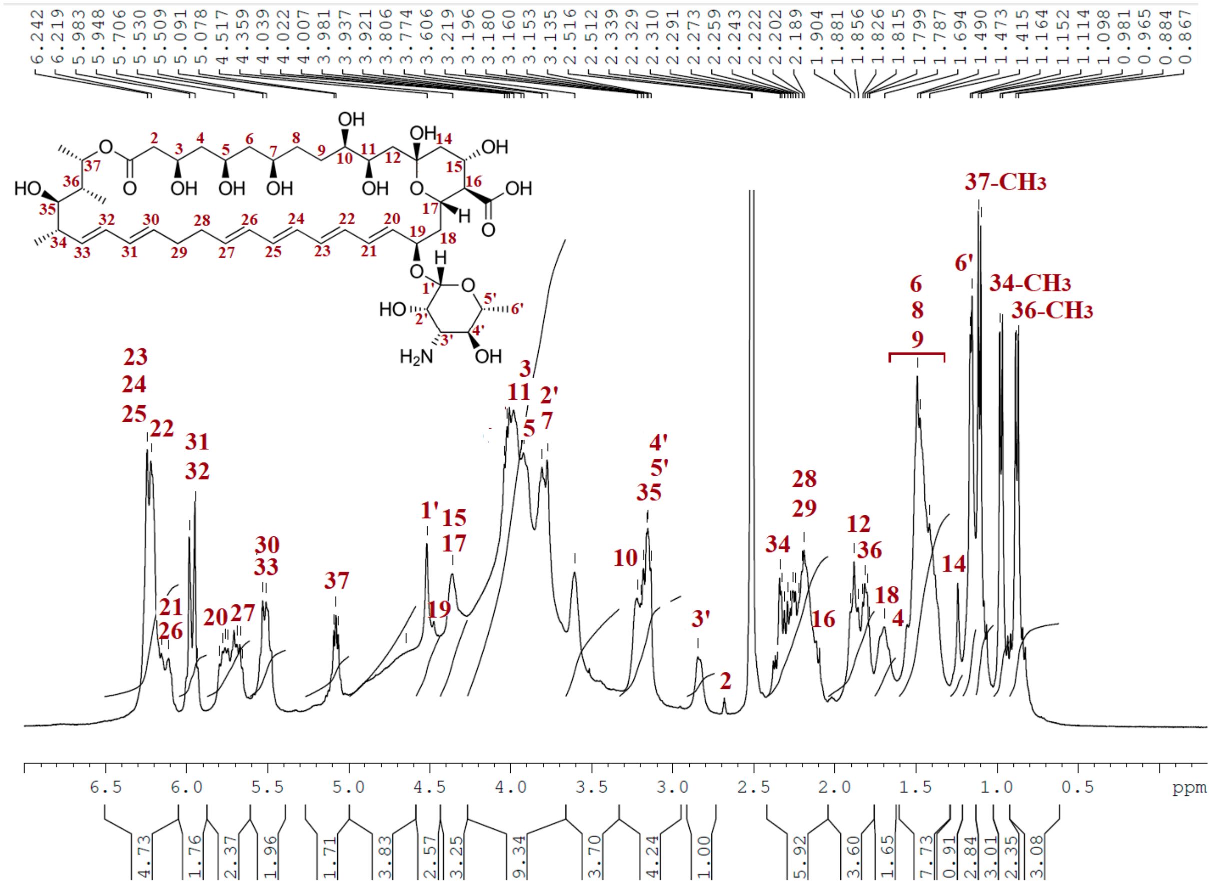


**Figure S3**. $^1$H-RMN spectrum of nystatin. IUPAC Name:(1S,3R,4R,7R,9R,11R,15S,16R,17R,18S,19E, 21E,25E, 27E,29E,31E,33R,35S,36R,37S)-33-[(2R,3S,4S,5S,6R)-4-amino-3,5-dihydroxy-6-methyloxan-2-yl]oxy-1,3,4,7,9,11,17,37-octahydroxy-15,16,18-trimethyl-13-oxo-14,39-dioxabicyclo[33.3.1]nonatriaconta-19,21,25,27,29,31-hexaene-36-carboxylic acid); signal attributions made according to ref. [44-46].

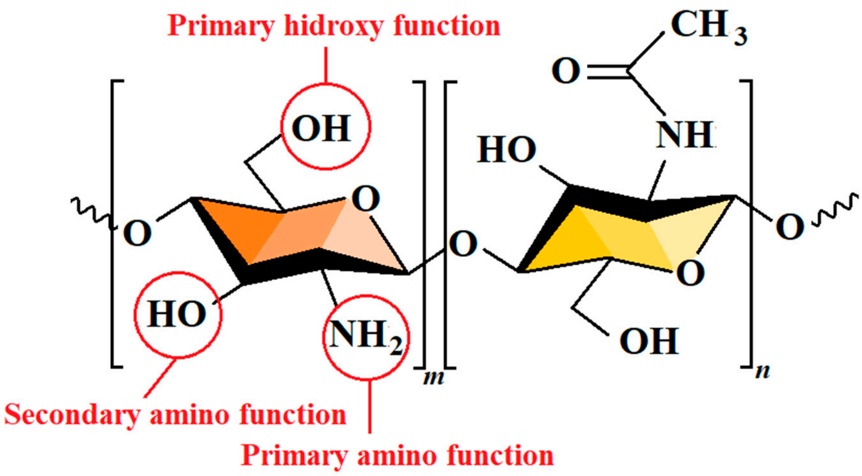


**Figure S4.** Chemical formula of chitosan with highlighting the functional groups: free primary amino groups ($NH_2$ to C2), primary hydroxyl group (OH to C6) and secondary hydroxyl groups (OH to C3); *m* and *n* represent the numbers of deacetylated (d-glucosamine) and N-acetyl-d-glucosamine repeating units, linked by (1-4)-β-glycosidic linkages, respectively.

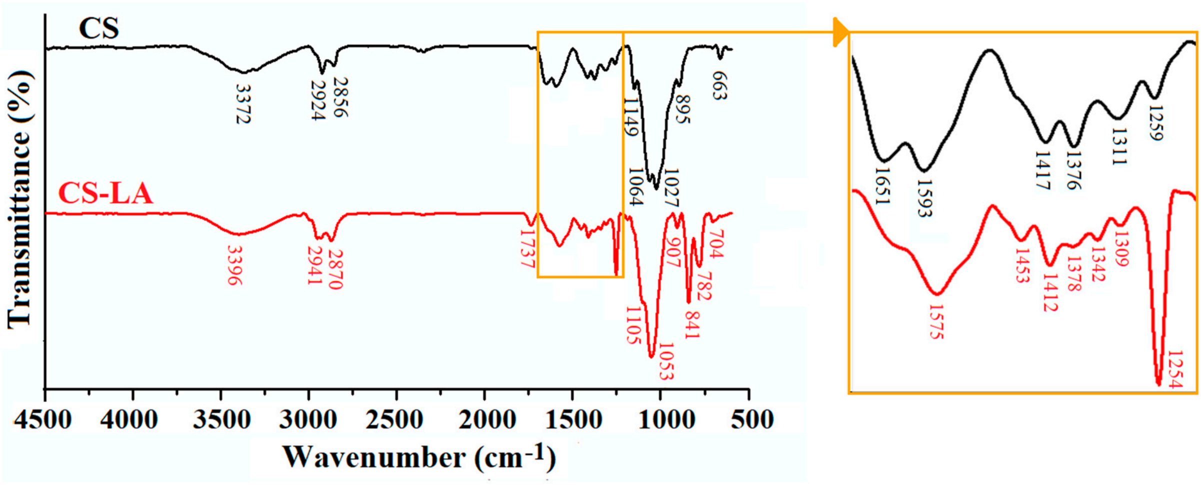


**Figure S5.** FTIR spectra of chitosan powder (CS) and chitosan gel (3%) after dispersion in 2% lactic acid solution (CS-LA).

The spectrum of chitosan shows polysaccharide characteristic absorption bands found in the range 4000-600 $cm^{-1}$: the broad band located between 3500-3100 $cm^{-1}$ results from the stretching vibrations of superimposed O–H and N–H bonds; the absorption bands corresponding to the asymmetric and symmetrical stretching vibrations of the C–H are observed at 2924 $cm^{-1}$, and 2856 $cm^{-1}$ respectively; the stretching vibration of the C=O bond of amide I (O=C–NHR) is distinguished at 1651 $cm^{-1}$; the band at 1593 $cm^{-1}$ is caused by the in-plane deformation vibration of the primary amine group $NH_2$ coupled with the stretching vibrations of amide II, and that at 1311 $cm^{-1}$ by the tensile vibrations of the C–N bond (amide III) ; the absorption bands given by the deformation vibrations of the $CH_2$ and $CH_3$ group appear at 1417 $cm^{-1}$, respectively 1376 $cm^{-1}$; the absorption band from 1149 $cm^{-1}$ is attributed to the asymmetric stretching vibrations of the C–O–C glycosidic bonds, and the bands from 1064 and 1027 $cm^{-1}$ are due to the C–O stretching vibrations, characteristic of the saccharide structure [19].

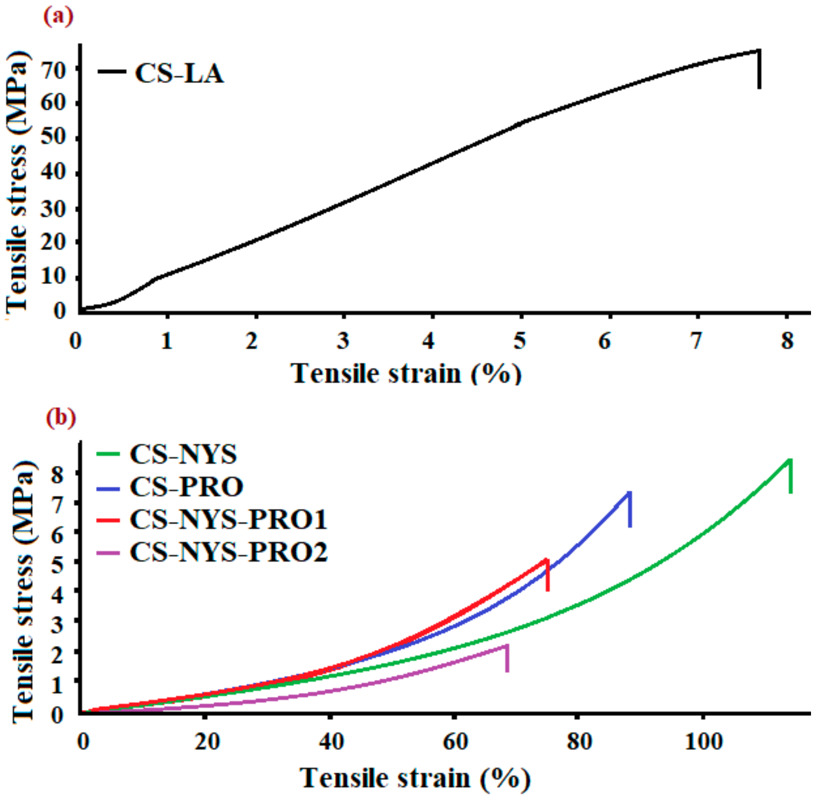


**Figure S6.** Characteristic stress-strain curves of the films: (**a**) without biological active compounds (CS-LA); (**b**) nystatin, propolis and nystatin/propolis loaded chitosan films (CS-NYS, CS-PRO, CS-NYS-PRO1, CS-NYS-PRO2).

**Table S1.** Parameters of pseudo-second order (PSO) and Korsmeyer-Peppas (K-P) kinetic models, where $k_s$ is the constant of the swelling rate, $S_e$ is the theoretical swelling capacity at equilibrium, $k_p$ is a constant dependent on the polymeric network; n is the diffusion parameter of aqueous PBS in the formulation film and SD represents the standard deviation of n=11); swelling capacity in PBS of chitosan charged films after 5 and 24 h.

| Film code | PSO model parameters | K-P model parameters | Swelling capacity after 5 h (%) | Swelling capacity after 24 h (%) |
|---|---|---|---|---|
| **CS-NYS** | $k_s = 1.86 \cdot 10^{-3}$<br>$Se = 600.74$<br>SD = 6.10 | $k_p = 450.17$<br>$n = 5.9 \cdot 10^{-2}$<br>SD = 8.03 | 618 | 751 |
| **CS-PRO** | $k_s = 5.29 \cdot 10^{2}$<br>$Se = 121.92$<br>SD = 0.37 | $k_p = 121.14$<br>$n = 4.87 \cdot 10^{-18}$<br>SD = 0.44 | 122 | 122 |
| **CS-NYS-PRO1** | $k_s = 1.21 \cdot 10^{-1}$<br>$Se = 130.84$<br>SD = 0.56 | $k_p = 129.14$<br>$n = 1.40 \cdot 10^{-3}$<br>SD = 0.71 | 129 | 129 |
| **CS-NYS-PRO2** | $k_s = 6.54 \cdot 10^{3}$<br>$Se = 119.35$<br>SD = 0.56 | $k_p = 117.93$<br>$n = 5.42 \cdot 10^{-18}$<br>SD = 0.71 | 118 | 118 |

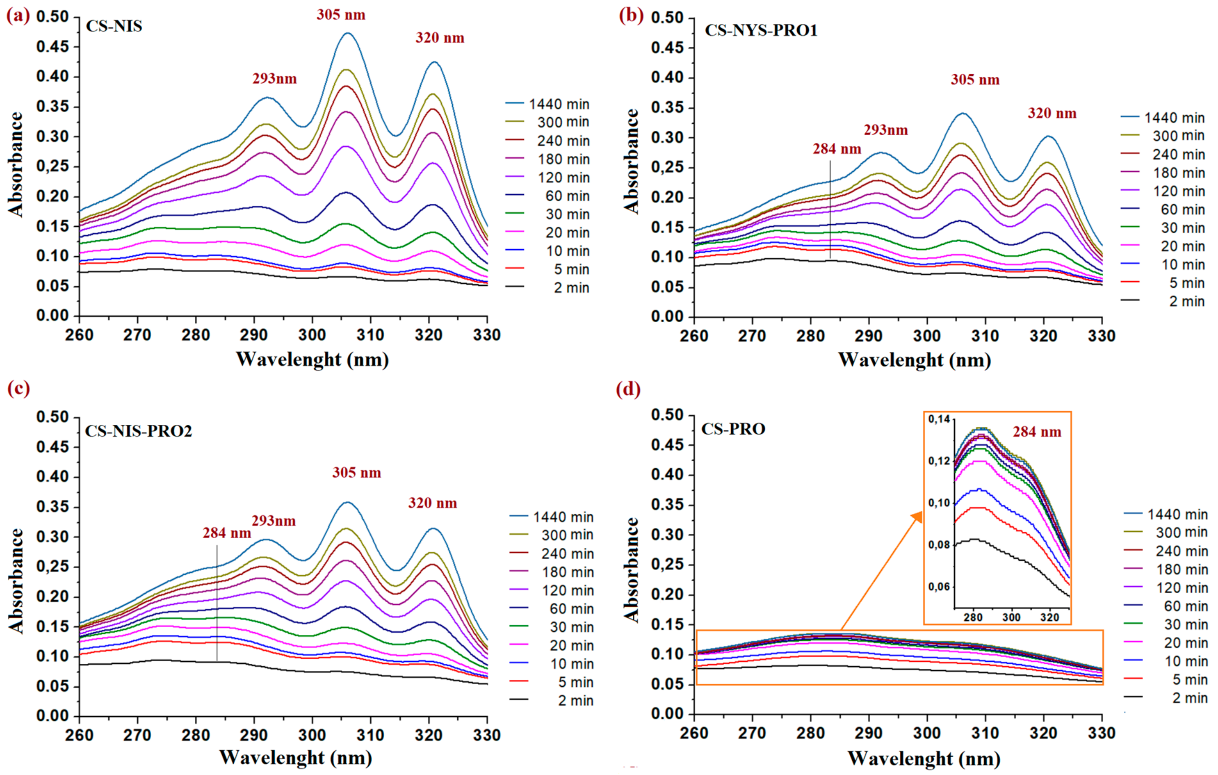


**Figure S7.** Evolution in time of UV-VIS spectra of: nystatin released from CS-NYS (**a**), CS-NYS-PRO1 (**b**) and CS-NYS-PRO2 (**c**); propolis released from CS-PRO (**d**).

**Table S2.** NYS and PRO release from chitosan films: Korsmeyer-Peppas kinetics parameters (k represents the transport constant, n is the diffusion exponent and SD is standard deviation of n=11), and release efficiency after 5 and 24 hours.

| Film code | Drug released | Korsmeyer-Peppas model parameters | Drug release efficiency after 5 hours (%) | Drug release efficiency after 1 day (%) |
|---|---|---|---|---|
| **CS-NYS** | NYS | $n$ = 0.4189821<br>$k$ = 0.045<br>SD = 0.37 | 48.6 | 55.2 |
| **CS-NYS-PRO1** | NYS | $n$ = 0.3209252<br>$k$ =0.072<br>SD = 0.50 | 45.9 | 53.3 |
| **CS-NYS-PRO2** | NYS | $n$ = 0.3098546<br>$k$ =0.071<br>SD = 0.34 | 42.8 | 48.7 |
| **CS-PRO** | PRO | $n$ = 0.0806324<br>$k$ = 0.157<br>SD = 0.35 | 24.2 | 24.2 |

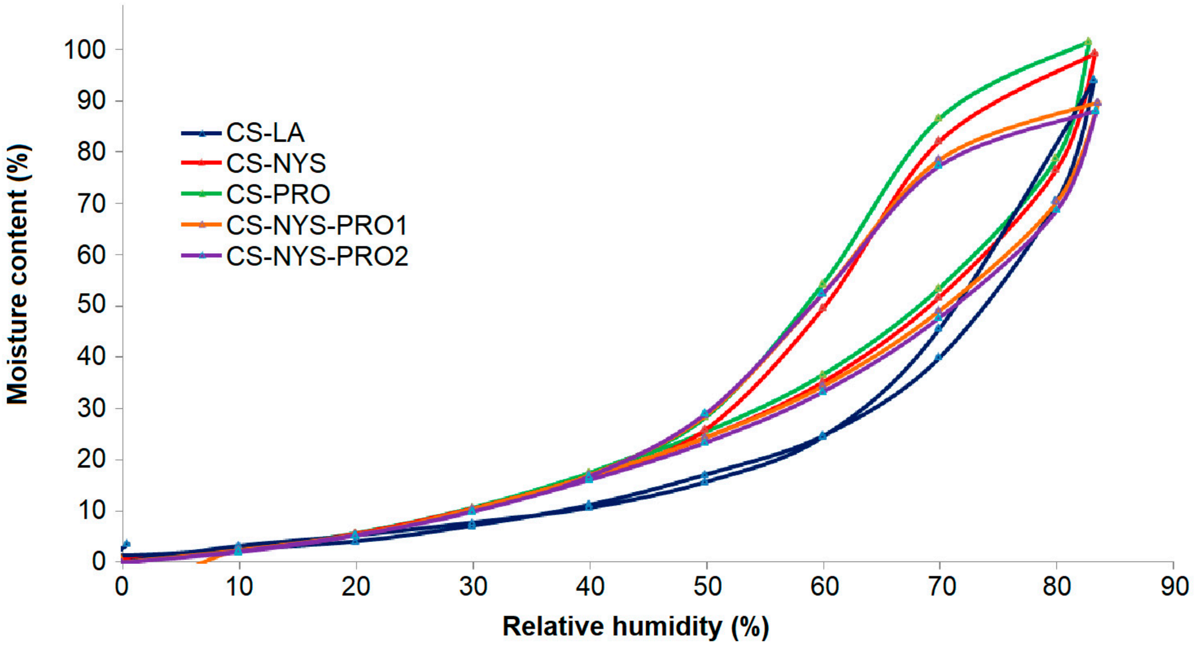


**Figure S8.** Sorption/desorption isotherms for the hydrogel formulations

**Table S3.** Surface parameters of the hydrogels evaluated based on adsorption/desorption isotherms: water vapor sorption capacity, final weight (W); average pore size ($r_{pm}$) and BET data (surface area and monolayer weight).

| Hydrogel Surface | W (%) | $r_{pm}$ (BJH model) (nm) | BET data * | |
|---|---|---|---|---|
| | | | Area (m²/g) | Monolayer (mg/g) |
| CS-LA | 94.1 | 6.8 | 275 | 788 |
| CS-NYS | 99.3 | 1.4 | 1379 | 399 |
| CS-PRO | 101.6 | 2.0 | 1035 | 294 |
| CS-NYS-PRO1 | 89.8 | 1.2 | 1444 | 412 |
| CS-NYS-PRO2 | 88.1 | 1.0 | 1727 | 492 |

Determined based on desorption branch of the isotherm (registered up to a relative humidity of 40 %)

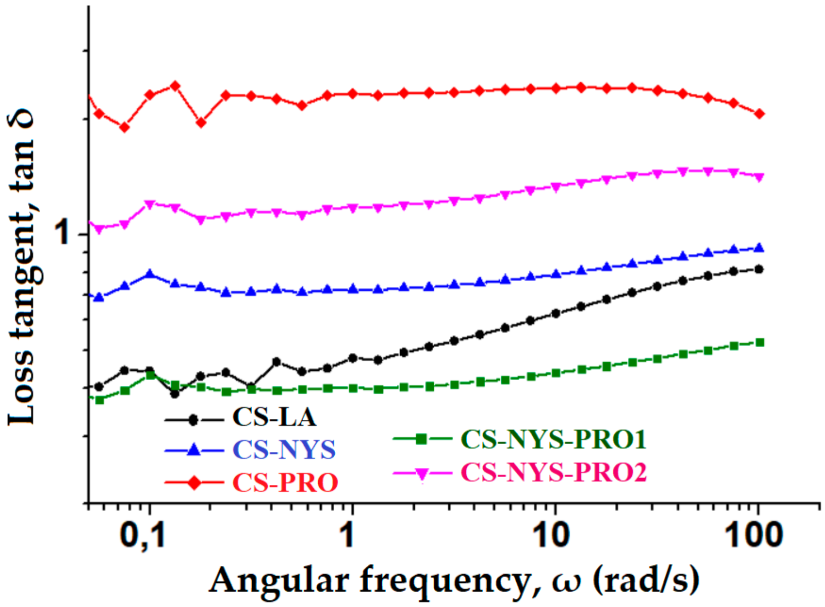


**Figure S9.** The loss tangent (tan δ = G'' / G') as a function of angular frequency.

**Table S4.** Viscoelastic parameters (G′, G′′ and tan δ) at a frequency of 1 Hz and viscous flow activation energy ($E_\eta$) calculated by fitting the temperature-dependence curve of the viscosity by Arrhenius equation.

| Gel formulation | G′ (Pa) | G′′ (Pa) | tan δ | $E_\eta$ (kcal/mol) |
|---|---|---|---|---|
| **CS-LA** | 42.64 | 20.30 | 0.47 | 5.18 |
| **CS-NYS** | 20.70 | 14.70 | 0.73 | 5.51 |
| **CS-PRO** | 0.94 | 2.22 | 2.31 | 5.67 |
| **CS-NYS-PRO1** | 107.05 | 43.96 | 0.40 | 4.81 |
| **CS-NYS-PRO2** | 4.43 | 5.45 | 1.18 | 5.28 |

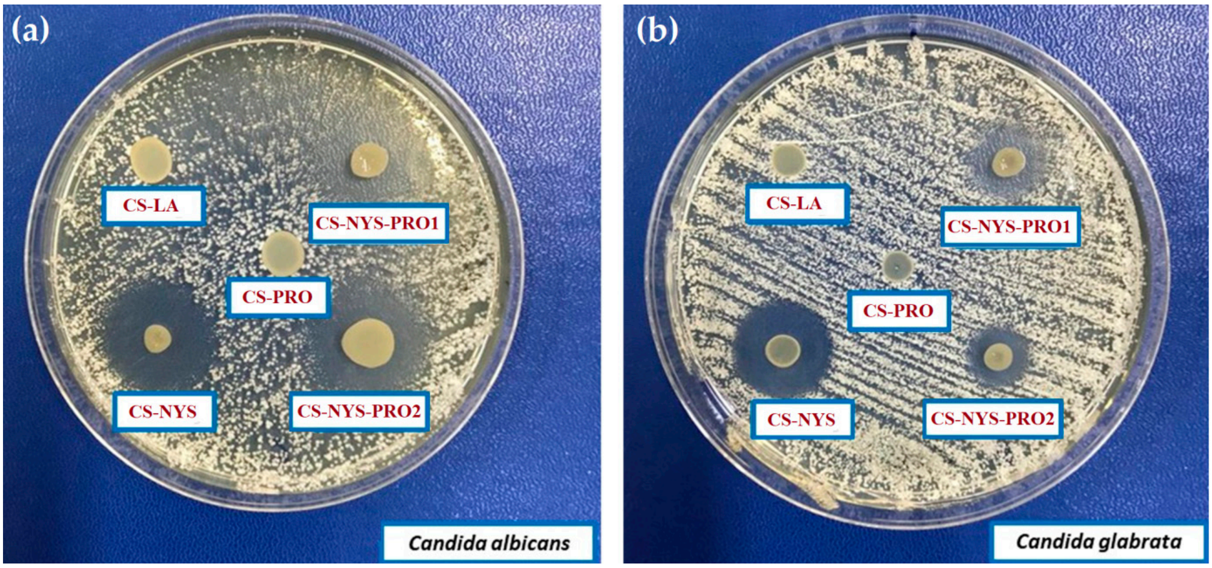


**Figure S10.** Antifungal activity of chitosan hydrogels against: (**a**) *C. albicans* and (**b**) *C. glabrata*

**Table S5.** Killing efficiency as a function of time for CS-NYS, CS-PRO and CS-NYS-PRO1 hydrogels, against *Candida albicans* in 24 h, as compared to the control test.

| **Time (hours)** | ***Candida albicans* (CFU/mL)** | | | |
|---|---|---|---|---|
| | **Control** | **CS-NYS** | **CS-PRO** | **CS-NYS-PRO1** |
| 0 | $1.24x10^7$ | $1.24x10^7$ | $1.24x10^7$ | $1.24x10^7$ |
| 6 | $1.64x10^7$ | $2.40x10^2$ | $7.60x10^6$ | $<10^1$ |
| 12 | $1.96x10^7$ | $<10^1$ | $3.00x10^6$ | $<10^1$ |
| 24 | $4.32x10^7$ | $<10^1$ | $2.28x10^6$ | $<10^1$ |
